# Rational Design of Constrained Peptides as Protein Interface Inhibitors

**DOI:** 10.3390/antib10030032

**Published:** 2021-08-16

**Authors:** Ramachandran Murali, Hongtao Zhang, Zheng Cai, Lian Lam, Mark Greene

**Affiliations:** 1 Cedars-Sinai Medical Center, Department of Biomedical Science, Research Division of Immunology, Los Angeles, CA 90211, USA; 2 Department of Pathology and Laboratory of Medicine, University of Pennsylvania, Philadelphia, PA 19104, USA; zhanghon@pennmedicine.upenn.edu (H.Z.); caizheng78@gmail.com (Z.C.); lian.lm4284@gmail.com (L.L.)

**Keywords:** ErbB, TNF, mimetic, receptor, protein-engineering, immunoadhesion, cancer

## Abstract

The lack of progress in developing targeted therapeutics directed at protein–protein complexes has been due to the absence of well-defined ligand-binding pockets and the extensive intermolecular contacts at the protein–protein interface. Our laboratory has developed approaches to dissect protein–protein complexes focusing on the superfamilies of erbB and tumor necrosis factor (TNF) receptors by the combined use of structural biology and computational biology to facilitate small molecule development. We present a perspective on the development and application of peptide inhibitors as well as immunoadhesins to cell surface receptors performed in our laboratory.

## 1. Introduction

Therapeutic development, particularly small molecule drug creation, has focused on enzymes. Other efforts have included natural compounds that are screened for the ability to induce phenotypic changes of transformed cells [1]. When human genome sequencing was undertaken and completed, expectations in the scientific community were that many more therapeutic targets might be identified. However, few “druggable” candidates were characterized [2,3,4]. This failure to identify new targets motivated many to develop new tools for “undruggable targets” [5,6,7,8,9,10].

Molecular assembly is a key step in many biological functions. Molecular ensemble formation is dynamic and can vary from simple dimerization to the assembly of large molecular complexes. Molecular ensemble formation may comprise protein-only, protein–DNA, or protein–lipid complexes. A variety of methods have been developed to target certain molecular complexes and include high throughput screening, phage display against protein complexes, and alteration of biological activities using antibodies and recombinant proteins [11,12,13].

Antibody still remains a preferred choice of therapeutic targeting agents especially by pharmaceutical enterprises. Size features affect certain functional aspects, including the lack of tissue penetration for imaging and crossing the blood–brain barrier. Humanization of therapeutic antibodies remains a time and resource consuming process. Peptide inhibitors are ideally suited to overcome these limitations and to create novel molecules beyond monoclonal antibodies. Furthermore, due to their small size, peptides are amenable for chemical modifications to attain clinically relevant pharmacological features, and their structural features may also facilitate small non-peptidic inhibitor development. 

Small peptides are ideal molecules to explore protein–protein interactions due to their intermediate size (1–2 kD), ease of synthesis, and ability to bind to protein surfaces. More and more peptides are now used in the clinic [14,15]. This progress is due to improvement in technologies including cost-effective large-quantity peptide synthesis and metabolic stability [16,17]. Increasingly, in addition to using peptides as therapeutics per se, novel approaches using peptide inhibitors are being adopted. These approaches include cell-permeable peptides [18], peptide-based cancer vaccines, and peptide-decorated nanoparticles for diagnostics and drug-delivery. Thus, peptide agents are versatile and can be adopted for diagnosis and therapy of diseases.

X-ray crystallography has helped to resolve molecular features of many protein complexes ranging from spherical viruses, cell surface receptors, intact antibodies, and (Fab)-antigen complexes at atomic resolution [19,20]. Based on the fundamental structural features of protein–protein complexes, we have developed a method to design and develop constrained peptide mimics specific for interaction surfaces using computational and structural biology approaches.

## 2. Antibody–Antigen Complex

Atomic structures of antibody–antigen complexes have aided the study of protein–protein interactions [21,22,23]. The Fab fragments of an antibody can recognize binding determinants such as small organic molecules (which includes haptens that can bind to larger proteins), carbohydrates, peptides, or large macromolecular complexes. Antibody–antigen complexes, in particular Fab–hapten complexes, have provided insight into aspects of molecular recognition [24]. Atomic analysis of antibody–hapten complexes has revealed that an antibody can recognize the three-dimensional structures of antigens. An antibody can distinguish between antigens of different enantiomers [25]. Thus, the ability of antibody to recognize “hot spots” on protein can be useful to identify critical residues in a putative targeted protein. 

We used these basic notions to study antibodies that bound to oncoproteins and to develop small peptidic loops from the antigen binding site that could act as small molecular peptidomimetics. To develop small peptides targeting the dimerization of Her2/neu receptor, we used the structure of a crystallographically resolved anti-Her2/neu antibody called Herceptin. Herceptin binds to the ectodomain of the HER2/neu protein. This antibody binding surface was used as a template to develop a small exocyclic peptide mimicking the antibody [26]. A detailed set of methods developing small antibody-like mimetics has been described previously [27]. 

In this review, we will present methodologies used in the development of peptide inhibitors that limit protein–protein interactions. We will discuss how peptide inhibitors are facile in developing therapeutic strategies. We focus on the development of antibody-like peptide mimics to cell surface receptors.

## 3. Structural Features of Protein–Protein Complexes

The enhanced understanding of protein structures at atomic resolution allowed Jones and Thornton [28] to propose a set of general parameters that govern protein–protein interaction. They identified three key features: (1) large protein–protein interface (>1000 Å^2^), (2) secondary structural elements (i.e., helix, beta-sheet, and flexible loops), and (3) molecular surface complementarity that included chemical/electrostatic charge and discrete geometrical features. 

The precise contribution of amino acids at the protein–protein interface through mutation studies expanded the concept of molecular recognition in terms of enthalpic parameters (i.e., polar and non-polar interactions such as hydrogen bond and hydrophobic contacts) to “shape complementarity” and “binding surface area” [29]. 

Large surfaces can be decorated with solvents which can contribute entropic components allowing weaker protein–protein interaction or enhanced enthalpic interactions by extending the hydrogen bond network [30,31,32,33]. Unlike enzyme–substrate complexes, structural understanding of protein–protein interfaces does not readily reveal critical contacts or a defined targetable site for developing small molecule ligands. These features have limited development of small molecules to target protein–protein/DNA interactions.

## 4. Receptor–Ligand Complex Peptide Inhibitor

### 4.1. Design Concept

A major impediment that limits targeting protein–protein interaction events is the lack of knowledge of key residues or regions involved in stabilizing the complex at the protein surface. Protein–protein interfaces involve large surfaces, multiple contact regions, and structural and geometrical constraints. Furthermore, protein–protein interactions or the interface is a dynamic solvent, and potential multiple partners can change the nature of the protein–protein complex. 

Our approach, as mentioned above, was guided by understanding of features of the antibody–antigen complex that showed: (1) the structure of a protein can be deconvoluted into scaffold (or framework region in antibody) and functional units (for example, the CDR regions in antibody). While some protein complexes may involve secondary structural elements such as alpha helices and beta strands, we focused on deciphering flexible loops in a protein complex as the critical functional units for the following reasons. We concentrated on efforts to create small exocyclic constrained peptide mimicking flexible loops [26,27,34,35], and we realized that a protein–protein interaction mediated by flexible loops tends to more dynamic. Our group incorporated the contributions of enthalpic and entropic components of the interaction which can be compensated either by solvents or weak interactions. Alpha helix and beta strand interactions are known to be influenced by enthalpic components which are affected by extensive hydrogen bonds and dipole interactions [36,37]. 

**Figure 1:**
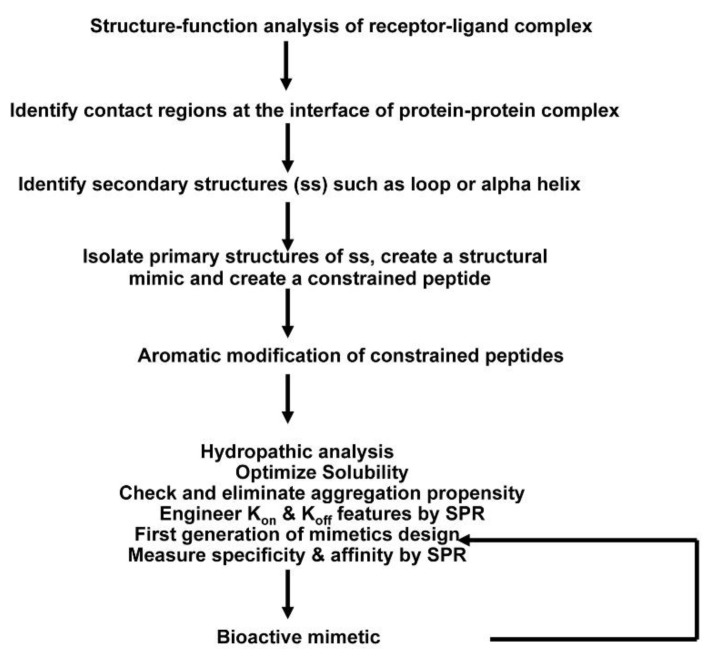
Overall scheme of the method used to create receptor complex peptide inhibitors. A systematic approach has been developed to design peptide inhibitors based on the three-dimensional structure of receptor–ligand complex, which involves peptide chemistry, computational biology, and use of surface plasmon resonance.

A general pathway scheme for the design and development of constrained exocyclic peptide targeting cell surface receptors is shown in Figure 1. Briefly, key contact residues required for stabilizing the receptor complex at the interface of the receptor–ligand complex are identified from the three-dimensional structure of the receptor complex. Then, the key residues are used to construct the peptide structure that can mimic the spatial disposition of the contact residues in the receptor/ligand. For in vivo stability, the peptides are further modified; peptide is cyclized through disulfide bonds followed by aromatic modification (i.e., additional residues Tyr/Phe are added at the N- and C-terminus of the peptide). Designed mimics are synthesized and screened for binding by SPR and in vitro assays. The most potent molecules are then tested in biological assays. In the following sections, structure-based approaches to design and develop peptide inhibitors of two receptor systems, ErbB and tumor necrosis factor receptor 1(TNFR1), are illustrated.

### 4.2. P185^neu/her2^ (Her2) Receptor Complex Targeted Peptidomimetics

The ErbB receptor superfamily consists of four members: EGFR, Her2/neu, Her3, and Her4. Except for Her2/neu, all the members are known to be activated by ligands. EGF and TGFβ can interact with EGFR. Neuregulin (NRG) can interact with Her3 and Her4.

**Figure 2:**
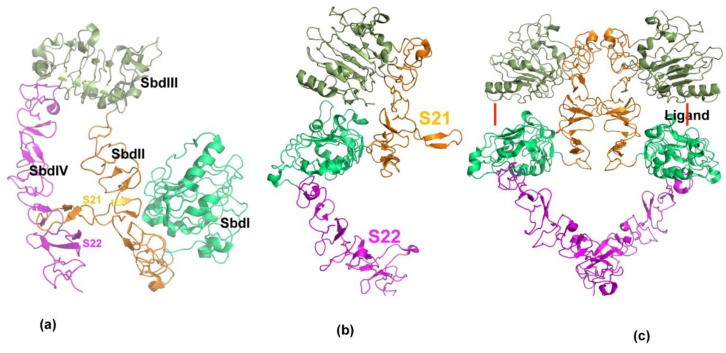
Structural features of erbB receptor activation. (**a**) Three-dimensional structure of ecto-domain of erbB receptor in resting state. Extracellular ErbB receptors consist of four distinct domains, SbdI–SbdIV. Each domain is shown in separate colors. In the resting state, the domains are held in closed or locked conformation through two long loops, S21 and S22 from domains SbdII and SbdIV. (**b**) Native state of Her2/neu is shown. In contrast to other members of erbB receptors, Her2 is observed only in an open or extended conformation. (**c**) Active state of erbB receptors is shown. Ligands binding to ErbB receptors induce dimer formation. Ligand binding site is shown by red line. ErbB dimers are stabilized by inter-locking loops near ligand-binding domain and at the membrane proximal (SbdIV) domains.

ErbB receptors are transmembrane proteins which possess extracellular or ligand binding domains, as well as transmembrane and tyrosine kinase domain in the cytoplasm. The extracellular domain consists of four subdomains including Leucine-rich repeat domains (SbdI and SbdIII) and cysteine-rich domains (SbdII and SbdIV) arranged in an alternate manner. Crystal structure studies of this family of receptors has revealed that the overall topology is conserved across ErbB receptors [38,39,40]. Furthermore, crystal structures show that Her2/neu exists as tethered [41] dimer and constitutively active. Other members undergo larger conformational rearrangement upon ligand binding for activation (formation of stable dimers) of the receptors [39]. The key feature of the erbB receptors is two long loops emanating from domain II (S21) and domain IV (S22) that exist in an extended state in Her2 and closed/open state in other erbB receptors; the closed state is considered inactive, and the extended state is considered as an active form (Figure 2). Notably, the Her2/neu receptor remains extended and is not known to exist in a closed form, suggesting that Her2/neu preferably forms a stable dimeric complex.

To develop dimeric inhibitors of the Her2 /neu associated ErbB receptor complex, we used the crystal structures of ErbB receptors [38,42] to create the small exocyclic peptide S22-AFA to target Her2-Her3 heterodimer formation [43]. This S22 peptide also blocked neuregulin mediated dimerization of the Her2-Her3 receptor complex. A dimeric erbB1-EGF model was developed, based on the existing experimental evidence that erbB1-EGF complex has a 2:2 stoichiometry [40]; the C-terminal part of subdomain IV is a dimeric interaction site [43]. Since the S22 loop stabilizes the active state of Her2-associated dimeric receptors, we focused on generating other peptides based on the structural features of S22.

**Figure 3:**
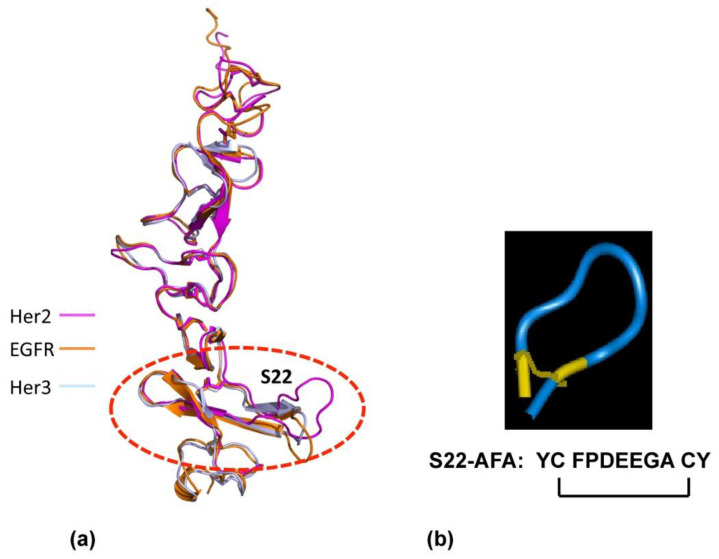
Design of ErbB dimerization inhibitor. (**a**) Structural similarity of the membrane-proximal domain SbdIV in EGFr, Her2, and Her3 is shown. (**b**) Designed molecular structure of S22-AFA is shown. Disulfide links are shown in yellow.

To verify whether the peptides derived from S22 display binding to erbB receptors, we studied the interaction of the S22-APE peptide by surface plasmon resonance (SPR) using a recombinant erbB2-SbdIV subdomain (Figure 3). Recombinant SbdIV bound to erbB2 with a reasonable affinity (K_D_, 1.89 μM). Subsequent screening of peptides showed that the S22-APE possessed a higher affinity binding to erbB1 (K_D_ 0.21 μM) than other peptides. Notably, the S22-AFA showed an overall comparable affinity to the three erbB receptors (ErbB1: K_D_ 0.41 μM, ErbB2: K_D_ 0.30 μM, and ErbB3: K_D_ 0.43 μM) and proved to be an effective inhibitor of erbB3-erbB2 and erbB3-erbB1 interactions in an SPR-based competition assay. S22-AFA inhibited T6-17 cell growth driven by Her2/neu receptors.

These studies demonstrated that (1) the identification of critical structural elements in a protein–protein interface is a key step for the development of peptide inhibitors, and (2) it is thought that small peptides are unlikely to completely prevent dimer formation due to their smaller surface, and small peptides can disrupt dimerization and mediate biological function by stabilizing a defective dimer complex.

### 4.3. TNFR Receptor Complex Targeted of Peptidomimetics

The tumor necrosis factor super family (TNFsf) consists of about 30 members, and the tumor necrosis factor receptor 1 (TNFR1) is the prototypical member of TNFRsf. Crystal structures of some members of TNFRsf have been resolved at atomic resolution; by homology, all the members share a common fold in the extra cellular domain-cystine-knot that is tandemly arranged from the N-terminus to the membrane proximal region [44,45]. The crystal structures of TNFR1 with and without TNFα have been determined [44,46,47] and revealed that the activation of TNFR1 by the ligand is not due to structural changes (i.e., ligand-induced conformational change) but by promoting receptor oligomerization as a trimer or higher ordered forms of trimers (e.g., hexamer).

**Figure 4:**
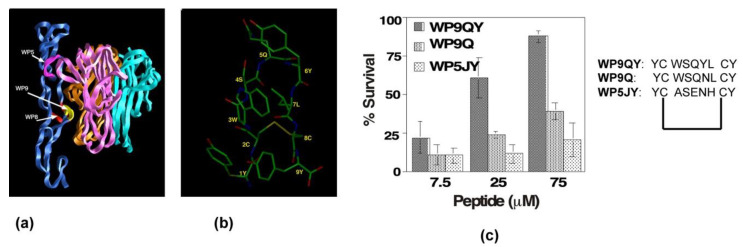
Design and development of anti-TNFR1 complex peptide inhibitor. (**a**) Three-dimensional structure of TNFR1-TNF receptor complex in ribbon representation. TNFR1 is shown in blue; each protomer of TNFα is shown in three-different colors. Three main contact loops, WP5, WP9, and WP8, involved in forming stable trimeric complex are indicted. The major contact, WP9, is highlighted in yellow. (**b**) Solution structure of WP9 is shown. (**c**) Inhibitory effects of peptides inhibitors derived from the structures of WP9 and WP5 loop in a cell survival assay. WP9QY inhibits TNFα-induced apoptosis in L929 cells. At 75 μM, WP9QY protected nearly 90% of cells from TNFα-induced cell death.

Our analysis of the crystal structure analysis of the TNFR1 and TNFα complex revealed that there were three distinct binding sites, termed “WP5, WP8 and WP9” [48] (Figure 4a). We developed and tested peptidomimetics derived from all three surface loops of the TNF receptor: loop (56–73) in domain 1; (76–83) loops of domain 2; and the first loop (107–114) of domain 3 (Figure 4a) on the TNF-receptor. The peptidomimetic engineered from the third domain (WP9QY: Tyr-Cys-Trp-Ser-Gln-Tyr-Leu-Cys-Tyr) inhibited TNFα binding (IC_50_ = 75 μM) to its receptor (Figure 4c). Additionally, the peptidomimetic protected cells against TNFα-induced cell death when apoptosis was induced with 7 pg of TNFα, suggesting that the peptide specifically binds to TNFα (Figure 4c). Kojima et al. showed that the peptidomimetic (WP9QY) reduced the clinical score of inflammation in a collagen-induced arthritis (CIA) mouse model [49], mimicking anti-TNF antibody function in reducing inflammation. This is one of the first peptide inhibitors, based on the structure of the TNFR1 receptor complex, targeting the receptor complex function. Subsequently, the methodology has been validated by developing peptide inhibitors to Fas-FasL and RANK receptor complexes [50,51].

## 5. Applications of Constrained Peptidomimetics and Creation of Immunoadhesins

Antibody engineering is an active area of research to replace or to create therapeutic antibody in a facile manner [52,53,54]. Conventional antibody generation involves immunizing animals with antigen, identification of therapeutic antibody, and creating monoclonal antibody species after affinity maturation. These processes are not only time-consuming but also resource intensive [55,56].

We have developed a method to engineer antibody-like binding proteins using our designed peptidic loops. The goal is to create a modular protein molecule where one or more structure-based constrained peptides can be tailored to create protein-scaffolds. These modular protein scaffolds, which we term “loop-bodies”, contain functional binding loops derived from receptors or antibodies and can include the Fc domain of immunoglobulin, and a supporting linker sequence around the loop to create novel immunoadhesins.

We chose to engineer the S22 peptide, which, as mentioned above, is derived from the HER2/neu receptor and interacts with all members of the ErbB receptors. By grafting the S22 peptide onto a unique linker sequence that is fused to Fc, we have observed greatly improved binding of the S22 sequence to HER2 as well as to EGFR and HER3. The in vivo activity of S22Fc loop bodies has been demonstrated by examining the effects on growing xenograft tumors. The same strategy can be expanded to establish a protein platform to produce a series of novel therapeutic proteins targeting receptors (e.g., EGFR, Her2, HER3, TNFR, RANK, etc.) or ligands (e.g., EGF and ligands for ErbB receptors, TNF, RANKL, etc.).

We have investigated several protein scaffolds as a platform to create more stable forms of the peptide. One scaffold is the streptavidin tetramer, which, when fused to the anti-p185*^her2/neu^* peptide AHNP, improved the association rate for binding to the target [52]. A second construct was developed by us using the Z domain of Staphylococcus to engineer proteins [57]. This class of novel proteins is an alternative to Fc fusion proteins and is able to interact with circulating IgGs while binding to a target antigen.

We have found a class of immunoadhesin that functioned well for the S22 species of peptides. The new S22-Fc species was developed to replace the combination of two different antibodies with one engineered fusion protein that can bind to multiple members of the ErbB receptors. The S22 was first designed to be expressed as a fusion protein with the intact Fc with both CH2 and CH3 domains from a human IgG1.

The Fc fusion protein was expressed in bacteria, in which glycosylation is deficient. It is reported that the aglycosylated Fc is very inefficient to bind to its receptors. Sazinsky et al. [58] described a T299A mutation near the glycosylation site (N297) that dramatically improved the interaction between aglycosylated Fc fragments and Fc receptors. We introduced this mutation to our recombinant protein that was expressed in *E. coli*.

We discovered that a short linker at the N-terminal improved the binding of the Fc fusion protein to receptors on T6-17 and NE91 cells. This novel short linker may represent a way to improve the stability of the construct for proper binding activity. The fusion protein produced from bacteria was named LS22FcT322G7.

Binding of the S22 peptide to ErbB receptors (EGFR, HER2, and HER3) has been determined with the dissociation constant (K_D_) in the 0.3–0.43 μM range [59]. We perform similar SPR assay to verify that the fusion protein, LS22FcT322G7, retains affinity for all these receptors.

**Figure 5:**
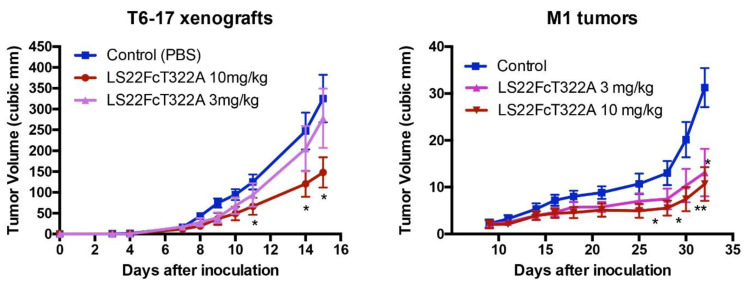
Inhibition of tumor growth. In the animal model of athymic mice carrying T6-17 or M1 xenografts, LS22FcT322A demonstrated dose-dependent activity. T6-17 and M1 tumors were established by subcutaneously implanting 5 × 10^4^ and 1 × 10^6^ cells, respectively, into the flank of nude mice. For T6-17 tumors, treatment started the next day after tumor inoculation. For M1 tumors, treatment started 10 days after. Mice were treated three times per week at two different dosages (3 mg/kg or 10mg/kg, i.p.). *t test* was performed to compare the difference between the size of tumors in the treated group and the control group. *: *p* < 0.05; **: *p* < 0.01.

We tested the activity of LS22FcT322G7 in the inhibition of in vivo tumor growth. Briefly, we studied two models: (1) T6-17 tumors, which comprise the murine tumor cell line that expresses human HER2/neu, and (2) M1 tumors, which comprise the murine tumor cell line driven by both EGFR and HER2. As shown in Figure 5, LS22FcT322G7 dose-dependently inhibits the tumor growth in both models. At the 10 mg/kg dosage, tumors in the treated group were very significantly smaller than those in the control groups.

## 6. Conclusions

We reviewed our approach to create protein–protein inhibitors targeting the erbB and TNF receptors. Our methods have utilized structural information that was combined with computational and biophysical tools. Additionally, we have shown that receptor complex peptide inhibitors are antibody-like protein molecules. Receptor peptide inhibitors developed by us are widely used by the research community, which has expanded our approach [60,61,62,63]. It is expected that these developments may lead to clinically viable candidates in future.

## Data Availability

Not applicable for a review article.

## References

[1] Newman D.J., Cragg G.M. (2012). Natural products as sources of new drugs over the 30 years from 1981 to 2010. J. Nat. Prod..

[2] Hopkins A.L., Groom C.R. (2002). The druggable genome. Nat. Rev. Drug Discov..

[3] Makley L.N., Gestwicki J.E. (2013). Expanding the number of ‘druggable’ targets: Non-enzymes and protein-protein interactions. Chem. Biol. Drug Des..

[4] Shendure J., Findlay G.M., Snyder M.W. (2019). Genomic Medicine-Progress, Pitfalls, and Promise. Cell.

[5] Hennemann H., Wirths S., Carl C. (2015). Cell-based peptide screening to access the undruggable target space. Eur. J. Med. Chem..

[6] Neklesa T.K., Winkler J.D., Crews C.M. (2017). Targeted protein degradation by PROTACs. Pharmacol. Ther..

[7] Ran X., Gestwicki J.E. (2018). Inhibitors of protein-protein interactions (PPIs): An analysis of scaffold choices and buried surface area. Curr. Opin. Chem. Biol..

[8] Ali A.M., Atmaj J., Van Oosterwijk N., Groves M.R., Dömling A. (2019). Stapled Peptides Inhibitors: A New Window for Target Drug Discovery. Comput. Struct. Biotechnol. J..

[9] Kessler D., Gmachl M., Mantoulidis A., Martin L.J., Zoephel A., Mayer M., Gollner A., Covini D., Fischer S., Gerstberger T. (2019). Drugging an undruggable pocket on KRAS. Proc. Natl. Acad. Sci. USA.

[10] Huang A., Garraway L.A., Ashworth A., Weber B. (2020). Synthetic lethality as an engine for cancer drug target discovery. Nat. Rev. Drug Discov..

[11] Cunningham A.D., Qvit N., Mochly-Rosen D. (2017). Peptides and peptidomimetics as regulators of protein-protein interactions. Curr. Opin. Struct. Biol..

[12] Cochran A.G. (2000). Antagonists of protein-protein interactions. Chem. Biol..

[13] Liu B.A., Engelmann B.W., Nash P.D. (2012). High-throughput analysis of peptide-binding modules. Proteomics.

[14] Lien S., Lowman H.B. (2003). Therapeutic peptides. Trends Biotechnol..

[15] Vlieghe P., Lisowski V., Martinez J., Khrestchatisky M. (2010). Synthetic therapeutic peptides: Science and market. Drug Discov. Today.

[16] Morrison C. (2018). Constrained peptides’ time to shine?. Nat. Rev. Drug Discov..

[17] Rastogi S., Shukla S., Kalaivani M., Singh G.N. (2019). Peptide-based therapeutics: Quality specifications, regulatory considerations, and prospects. Drug Discov. Today.

[18] Nagai Y., Lam L., Greene M.I., Zhang H. (2019). FOXP3 and Its Cofactors as Targets of Immunotherapies. Engineering.

[19] Burley S.K., Berman H.M., Kleywegt G.J., Markley J.L., Nakamura H., Velankar S. (2017). Protein Data Bank (PDB): The Single Global Macromolecular Structure Archive. Methods Mol. Biol..

[20] Berman H.M., Vallat B., Lawson C.L. (2020). The data universe of structural biology. IUCrJ.

[21] Jones S., Thornton J.M. (1996). Principles of protein-protein interactions. Proc. Natl. Acad. Sci. USA.

[22] Sundberg E.J., Mariuzza R.A. (2002). Molecular recognition in antibody-antigen complexes. Adv. Protein Chem..

[23] Lafont V., Schaefer M., Stote R.H., Altschuh D., Dejaegere A. (2007). Protein-protein recognition and interaction hot spots in an antigen-antibody complex: Free energy decomposition identifies “efficient amino acids”. Proteins.

[24] Strong R.K., Campbell R., Rose D.R., Petsko G.A., Sharon J., Margolies M.N. (1991). Three-dimensional structure of murine anti-p-azophenylarsonate Fab 36-71. 1. X-ray crystallography, site-directed mutagenesis, and modeling of the complex with hapten. Biochemistry.

[25] Parkkinen T., Nevanen T.K., Koivula A., Rouvinen J. (2006). Crystal structures of an enantioselective fab-fragment in free and complex forms. J. Mol. Biol..

[26] Park B.W., Zhang H.T., Wu C., Berezov A., Zhang X., Dua R., Wang Q., Kao G., O’Rourke D.M., Greene M.I. (2000). Rationally designed anti-HER2/neu peptide mimetic disables P185HER2/neu tyrosine kinases in vitro and in vivo. Nat. Biotechnol..

[27] Murali R., Greene M.I. (2012). Structure based antibody-like peptidomimetics. Pharmaceuticals.

[28] Jones S., Marin A., Thornton J.M. (2000). Protein domain interfaces: Characterization and comparison with oligomeric protein interfaces. Protein Eng..

[29] Vaughan C.K., Buckle A.M., Fersht A.R. (1999). Structural response to mutation at a protein-protein interface. J. Mol. Biol..

[30] Bhat T.N., Bentley G.A., Boulot G., Greene M.I., Tello D., Dall’Acqua W., Souchon H., Schwarz F.P., Mariuzza R.A., Poljak R.J. (1994). Bound water molecules and conformational stabilization help mediate an antigen-antibody association. Proc. Natl. Acad. Sci. USA.

[31] Braden B.C., Poljak R.J. (1995). Structural features of the reactions between antibodies and protein antigens. FASEB J..

[32] Yan C., Wu F., Jernigan R.L., Dobbs D., Honavar V. (2008). Characterization of protein-protein interfaces. Protein J..

[33] Visscher K.M., Kastritis P.L., Bonvin A.M. (2015). Non-interacting surface solvation and dynamics in protein-protein interactions. Proteins.

[34] Kieber-Emmons T., Murali R., Greene M.I. (1997). Therapeutic peptides and peptidomimetics. Curr. Opin. Biotechnol..

[35] Murali R., Greene M.I. (1998). Structure-based design of immunologically active therapeutic peptides. Immunol. Res..

[36] von Grafenstein S., Wallnoefer H.G., Kirchmair J., Fuchs J.E., Huber R.G., Schmidtke M., Sauerbrei A., Rollinger J.M., Liedl K.R. (2015). Interface dynamics explain assembly dependency of influenza neuraminidase catalytic activity. J. Biomol. Struct. Dyn..

[37] Watkins A.M., Wuo M.G., Arora P.S. (2015). Protein-Protein Interactions Mediated by Helical Tertiary Structure Motifs. J. Am. Chem. Soc..

[38] Franklin M.C., Carey K.D., Vajdos F.F., Leahy D.J., de Vos A.M., Sliwkowski M.X. (2004). Insights into ErbB signaling from the structure of the ErbB2-pertuzumab complex. Cancer Cell.

[39] Garrett T.P., McKern N.M., Lou M., Elleman T.C., Adams T.E., Lovrecz G.O., Kofler M., Jorissen R.N., Nice E.C., Burgess A.W. (2003). The crystal structure of a truncated ErbB2 ectodomain reveals an active conformation, poised to interact with other ErbB receptors. Mol. Cell.

[40] Garrett T.P., McKern N.M., Lou M., Elleman T.C., Adams T.E., Lovrecz G.O., Zhu H.J., Walker F., Frenkel M.J., Hoyne P.A. (2002). Crystal structure of a truncated epidermal growth factor receptor extracellular domain bound to transforming growth factor alpha. Cell.

[41] Cho H.S., Leahy D.J. (2002). Structure of the extracellular region of HER3 reveals an interdomain tether. Science.

[42] Cho H.S., Mason K., Ramyar K.X., Stanley A.M., Gabelli S.B., Denney D.W., Leahy D.J. (2003). Structure of the extracellular region of HER2 alone and in complex with the Herceptin Fab. Nature.

[43] Berezov A., Chen J., Liu Q., Zhang H.T., Greene M.I., Murali R. (2002). Disabling receptor ensembles with rationally designed interface peptidomimetics. J. Biol. Chem..

[44] Naismith J.H., Devine T.Q., Kohno T., Sprang S.R. (1996). Structures of the extracellular domain of the type I tumor necrosis factor receptor. Structure.

[45] Naismith J.H., Sprang S.R. (1998). Modularity in the TNF-receptor family. Trends Biochem. Sci..

[46] Baeyens K.J., De Bondt H.L., Raeymaekers A., Fiers W., De Ranter C.J. (1999). The structure of mouse tumour-necrosis factor at 1.4 A resolution: Towards modulation of its selectivity and trimerization. Acta Cryst. D Biol. Cryst..

[47] Ono M., Horita S., Sato Y., Nomura Y., Iwata S., Nomura N. (2018). Structural basis for tumor necrosis factor blockade with the therapeutic antibody golimumab. Protein Sci..

[48] Takasaki W., Kajino Y., Kajino K., Murali R., Greene M.I. (1997). Structure-based design and characterization of exocyclic peptidomimetics that inhibit TNF.alpha. binding to its receptor. Nat. Biotechnol..

[49] Kojima T., Aoki K., Nonaka K., Saito H., Azuma M., Iwai H., Varghese B.J., Yoshimasu H., Baron R., Ohya K. (2005). Subcutaneous injections of a TNF-alpha antagonistic peptide inhibit both inflammation and bone resorption in collagen-induced murine arthritis. J. Med. Dent. Sci..

[50] Cheng X., Kinosaki M., Takami M., Choi Y., Zhang H., Murali R. (2004). Disabling of receptor activator of nuclear factor-kappaB (RANK) receptor complex by novel osteoprotegerin-like peptidomimetics restores bone loss in vivo. J. Biol. Chem..

[51] Hasegawa A., Cheng X., Kajino K., Berezov A., Murata K., Nakayama T., Yagita H., Murali R., Greene M.I. (2004). Fas-disabling small exocyclic peptide mimetics limit apoptosis by an unexpected mechanism. Proc. Natl. Acad. Sci. USA.

[52] Masuda K., Richter M., Song X., Berezov A., Masuda K., Murali R., Greene M.I., Zhang H. (2006). AHNP-streptavidin: A tetrameric bacterially produced antibody surrogate fusion protein against p185her2/neu. Oncogene.

[53] Mitran B., Andersson K.G., Lindström E., Garousi J., Rosestedt M., Tolmachev V., Ståhl S., Orlova A., Löfblom J. (2019). Affibody-mediated imaging of EGFR expression in prostate cancer using radiocobalt-labeled DOTA-ZEGFR:2377. Oncol. Rep..

[54] Simeon R., Chen Z. (2018). In vitro-engineered non-antibody protein therapeutics. Protein Cell.

[55] Hanack K., Messerschmidt K., Listek M. (2016). Antibodies and Selection of Monoclonal Antibodies. Adv. Exp. Med. Biol..

[56] Thakur A., Huang M., Lum L.G. (2018). Bispecific antibody based therapeutics: Strengths and challenges. Blood Rev..

[57] Cai Z., Fu T., Nagai Y., Lam L., Yee M., Zhu Z., Zhang H. (2013). scFv-based “Grababody” as a general strategy to improve recruitment of immune effector cells to antibody-targeted tumors. Cancer Res..

[58] Sazinsky S.L., Ott R.G., Silver N.W., Tidor B., Ravetch J.V., Wittrup K.D. (2008). Aglycosylated immunoglobulin G1 variants productively engage activating Fc receptors. Proc. Natl. Acad. Sci. USA.

[59] Berezov A., Zhang H.T., Greene M.I., Murali R. (2001). Disabling erbB receptors with rationally designed exocyclic mimetics of antibodies: Structure-function analysis. J. Med. Chem..

[60] Kato G., Shimizu Y., Arai Y., Suzuki N., Sugamori Y., Maeda M., Takahashi M., Tamura Y., Wakabayashi N., Murali R. (2015). The inhibitory effects of a RANKL-binding peptide on articular and periarticular bone loss in a murine model of collagen-induced arthritis: A bone histomorphometric study. Arthritis Res. Ther..

[61] Ding H., Gangalum P.R., Galstyan A., Fox I., Patil R., Hubbard P., Murali R., Ljubimova J.Y., Holler E. (2017). HER2-positive breast cancer targeting and treatment by a peptide-conjugated mini nanodrug. Nanomedicine.

[62] Haque Bhuyan M.Z., Tamura Y., Sone E., Yoshinari Y., Maeda C., Takahashi M., Tabata Y., Murali R., Waki Y., Aoki K. (2017). The intra-articular injection of RANKL-binding peptides inhibits cartilage degeneration in a murine model of osteoarthritis. J. Pharmacol. Sci..

[63] Idress M., Milne B.F., Thompson G.S., Trembleau L., Jaspars M., Houssen W.E. (2020). Structure-Based Design, Synthesis and Bioactivity of a New Anti-TNFα Cyclopeptide. Molecules.

